# Accuracy of self-perceived risk perception of breast cancer development in Iranian women

**DOI:** 10.1186/s12905-021-01238-z

**Published:** 2021-03-04

**Authors:** Karimollah Hajian-Tilaki, Maryam Nikpour

**Affiliations:** 1 Department of Biostatistics and Epidemiology, Babol University of Medical Sciences, Babol, Iran; 2 Social Determinants of Health Research Center, Health Research Institute, Babol University of Medical Sciences, Babol, Iran; 3 Non-Communicable Disease Research Center, Health Research Institute, Babol University of Medical Sciences, Babol, Iran

**Keywords:** Self-perceived risk, Actual risk, Five-year risk, Lifetime risk, Gail’s model

## Abstract

**Background:**

The accuracy of subjective risk perception is a matter of concern in breast cancer development. The objective of this study was to evaluate the accuracy of self-perceived risk assessment of breast cancer development and compared to actual risk in Iranian women.

**Methods:**

The demographic, clinical, and reproductive characteristics of 800 women aged 35–85 years were collected with an in-person interview. The self-perceived risk and the actual risk were assessed using the visual analog scale (VAS) and he Gail model respectively. Gail’s cutoff of 1.66% risk was used to categorize the estimated 5-year actual risk as low/average risk (< 1.66%) and high risk (≥ 1.66). In low/average risk, if the self-perceived risk > actual risk, then individuals were considered as overestimating. Similarly, in high-risk women, if the perceived risk < actual risk, then, the subjects were labeled as under-estimate; otherwise, it was labeled as accurate. The Kappa statistics were used to determine the agreement between self-perceived risk and actual risk. ROC analysis was applied to determine the accuracy of self-perceived risk in the prediction of actual risk.

**Results:**

The perceived risk was significantly higher than actual risk (*p* = 0.001, 0.01 for 5-year and lifetime risk respectively). Both in low and high-risk groups about half of the women over-estimate and underestimate the risk by subjective risk perception. For a 5-year risk assessment, there was no agreement between perceived risk and actual risk (Kappa = 0.00, *p* = 0.98) but a very low agreement between them in lifetime risk assessment (Kappa = 0.09, *p* = 0.005). The performance of accuracy of risk perception versus actual risk was very low (AUC = 0.53, 95% CI 0.44–0.61 and AUC = 0.58, 95% CI 0.54–0.62 for the 5-year risk and lifetime risk respectively).

**Conclusion:**

The clinical performance of risk perception based on VAS is very poor. Thus, the efforts of the public health education program should focus on the correct perception of breast cancer risk among Iranian women.

## Background

Breast cancer (BC) is the most common cancer [1] and the second cause of death (after lung cancer) in women worldwide [2]. BC is one of the most expensive cancers in the world with a financial burden of about $ 88 billion annually [3]. In Iran, BC is the most common cancer and it is composed of a quarter of malignancies. The age of involvement by breast cancer in Iranian women is about 10 years less than other developed countries [3].

Incidence and mortality rates of BC are increasing in Iranian women [4, 5]. The increasing incidence in developing counties mainly attributed to changes in lifestyles and reproductive behaviors [6–10]. The majority of women with BC are diagnosed at a higher stage of the disease that is corresponded with a high mortality rate [11–13]. To establish a preventive strategy for early detection, the risk assessment is primarily interested in BC from a public health perspective that can be used as a preventive strategy for early detection. Several methods of risk assessment of BC have been suggested [14–17]. They can be categorized as objective risk assessment or actual risk and subjective risk perception. The Gail model was mainly adopted for quantitative assessment of BC development. It predicts the actual risk as an objective measure based on the profile of risk factors [15]. The second method is the self-perceived risk perception that is based on the subjective matter on the visual analog scale (VAS). Another method of perceived risk assessment is the health belief model (HBM) scale to measure the perceived risk in different domains (susceptibility, seriousness, benefit, barriers, self-efficacy/confidence, and health motivation) [17]. The performance of HBM on screening behaviors has been acknowledged in several investigations [13, 17–19].

The self-perceived risk perception based on the analog scale is a simple method that may use in clinical practices for purpose of screening. It can be motivates the women to undergo the screening program such as breast self- examination (BSE), breast clinical examination (BCE) and mammography. However, subjective risk perception is likely to be either optimistic or pessimistic [20–23]. Thus, inaccurate risk perception may induce psychological symptoms, in particular in the case of over-estimate. It may concern that women either underestimate or overestimate their risk based on this subjective matter. Meanwhile, the subjective risk assessment depends on the culture and environmental conditions. Its accuracy is a matter of concern, and data in this regard is sparse in the Asian population, particularly in the Islamic Republic of Iran. This study aimed to evaluate the accuracy of self-perceived risk assessment compared to the objective risk and to identify characteristics of individuals who do not accurately perceive their risk of developing BC.

## Methods

### Study design and subjects

This cross-sectional study was conducted in Babol, northern Iran in 2018. Data were reanalyzed for risk assessment of breast cancer development. A total of 800 women at age of 35–85 years participated in the study. In comparing between the mean of objective risk by Gail model and perceived risk by VAS scale in our pair samples, the allocated sample size could detect the effect size of 0.1 with 95% confidence interval and 80% power. A cluster sampling technique was used to recruit individuals in the study in the randomly selected community-based clusters and also the outpatient clinic. The details of sampling methods were described elsewhere [12]. In brief, 20 randomly selected community-based clusters and 3 outpatient clinics in the major educational hospitals were used to select samples. Individuals with a previous history of a histologically confirmed diagnosis of breast cancer were excluded. The study protocol was approved by the institutional board of the National Institute of Medical Research Development (NIMAD), Tehran, Iran.

### Data and instruments

The data of demographic, clinical and reproductive characteristics were collected with in-person interviews using a standard questionnaire of breast cancer assessment. This questionnaire was developed by US National Cancer Institute and it is included patient eligibility, demographic and para-clinical findings of biopsy and family history [24]. More specifically, these data composed of age, educational level, marital status and occupation, the first menstrual age, age at first childbirth. Also there are information such as history of breast cancer in close family members (mother, daughters, and sisters), history of biopsy of the breast, number of biopsies, identify of dysplasia in the biopsy. We also used the Persian version of the breast cancer awareness measure (BCAM) questionnaire [25]. This scale originally developed by UK cancer center and validated specific to BC awareness [26]. The Persian version of this scale was investigated by Heidari and Feizi [25] that showed a high test–retest reliability (ICC = 0.84) and internal consistency (Cronbach’s alpha = 0.88). This scale composed of the knowledge of women in breast cancer risk factors and the sign and symptoms of breast cancer. It has 14 items of multiple-choice scale to measure the awareness of risk factors and 8-item scale to assess the signs and symptoms with binary response. The score of both measures was categorized as low (≤ the average scale) and high (> average of scale). The self-perceived 5-year and lifetime risk were assessed using a visual analog scale (VAS) ranging from 0 to 100. The participants showed the appropriate point in this scale for their risk perception of breast development for the next 5-years and also lifetime risk. Meanwhile, the objective risk/actual risk was calculated from the modified Gail’s model. This model was automated with an interactive online program based on clinical and reproductive data of individuals’ risk profiles. This program computes the actual 5-year and lifetime risk and also the average risk with similar age of individuals in the population of women. The details of this risk calculation were explained elsewhere [12].

### Statistical analysis

We used SPSS software version 18.0 for data analysis. The descriptive statistics for quantitative data of self-perceived and actual risk were shown as median and quartiles and for categorical data as frequencies and percentages. The Wilcoxon related sample test was used to compare the mean of perceived risk and actual risk. The Gail’s cutoff criterion of 1.66% risk was used to categorize the estimated 5-year risk/actual risk of participants as low/average risk (< 1.66%) and high risk (≥ 1.66). A similar classification was applied for lifetime risk at a cutoff point of 10% (< 10% vs. ≥ 10% risk), According to objective risk status (low/average and high risk), among the participants with low/average risk, if the self-perceived risk > actual risk, then the perceived risk was categorized as over-estimate. While among individuals with high actual risk, if the perceived risk < actual risk, then, the subjects were as under-estimate; otherwise, it was labeled as accurate. The frequencies and percentage of over-estimate and under-estimate of self-perceived risk was calculated according to actual risk status. The association of individuals’ characteristics with over-estimate and under-estimate of self-perceived risk was determined by the cross-classification of data using the Chi-square test. Based on the categorization of both perceived risk and actual risk (cutoff value of 1.66% of the 5-year risk and 10% of lifetime risk), the Kappa statistics as an index of agreement and the *p* value of McNemar test was calculated. Moreover, ROC analysis was applied to identify the diagnostic value of perceived risk to classify correctly the actual risk based on the cutoff points of 1.66% and 10% for actual 5-year risk and actual lifetime risk respectively. The area under the curve and its *p* value were calculated. All tests were two-sided and a *p* value less than 0.05 was considered as significant level.

## Results

The mean age (SD) of participants was 47.63 (10.46) years and the majority of individuals were at age 35–49 years (61.5%) and the minority (7.1%) was at age 65–85 and the rest were at age of 50–64 years. The majority of women were at an educational level of high school or higher (81.0%) and housewife (74.1%). A few participants (7%) had a family history of breast cancer in first-degree relatives. Almost, 84.9% were married and 6.6% single and the remainders were either divorced or widow (8.5%). About half of participants had a low level of knowledge of breast cancer risk factors and 34.8% had a low level of awareness of signs and symptoms of breast (Table 1). Table 2 shows that the mean of self-reported perceived 5-year risk and lifetime risk was significantly higher than actual risk (9.19 ± 16.1 vs. 0.89 ± 0.89, *p* = 0.001, and 14.87 ± 20.79 vs. 8.87 ± 3.84 vs. *p* = 0.01 respectively). While the median of perceived risk was rather lower than actual risk (0% vs. 0.7%, and 5.0% vs. 8.3%). But the third quartile (Q3) of perceived risk much greater than the actual risk for both 5-year and lifetime risk. Table 3 indicates that among participants with low or average 5-year risk for disease, roughly 45.7% of women, their perceived risk was over-estimated. While among high risk, 54.1% of participants under-estimated in their perceived risk. Based on the objective/actual 5-year risk assessment (Gail criteria) 61 women (7.5%) were at high risk. In contrast, for perceived risk with a similar cutoff value was 366 (45.8%). This also shows that higher level 5-year perceived risk compared to the actual risk for BC. The Kappa statistics across data in Table 3 show that there is no agreement between perceived risk and actual risk (Kappa = 0.00, *p* = 0.98).

**Table 1:** Characteristics of study subjects

Characteristics	N	%
Age (year)		
35–49	492	61.5
50–64	251	31.4
≥ 65	57	7.1
1st menstrual age (year)		
< 12	119	15.1
≥ 12	667	84.9
Age at 1st birth (year)		
< 20	249	33.2
20–24	317	42.3
25–29	141	18.8
≥ 30	43	5.7
Breast cancer at 1st degree of relatives		
No	742	93.0
Yes	58	7.0
No of biopsy		
None/not applicable	744	93.0
≥ 1	56	7.0
Education		
Illiterate	78	9.8
Primary	154	19.3
High school	393	49.1
University	175	21.9
Occupation		
Housewife	593	74.0
Employee	175	21.9
Retired	32	4.0
Marital status		
Single	53	6.6
Married	679	84.9
Divorced/widow	68	8.5
Knowledge of risk factor		
Low	419	52.6
High	377	47.4
Awareness of signs and symptoms of BC		
Low	275	34.8
High	516	65.2

**Table 2:** The mean (SD) and the quartiles of self-perceived risk and estimated actual risk of women BC development in study sample

	Perceived 5-year risk	Perceived lifetime risk	Actual 5-year risk	Actual lifetime risk
Mean (SD) %	9.19 (16.1)	14.87 (20.79)	0.89 (0.89)	8.87 (3.84)
Q1%	0.00	0.00	0.50	6.82
Q2 (Median) %	0.00	5.00	0.70	8.30
Q3%	10.00	20.00	1.10	10.30

**Table 3:** The self-perceived 5-year risk of BC development according to corresponded estimated actual 5-year risk

Perceived 5-year risk	Actual 5-year risk		All
	< 1.66% risk	≥ 1.66% risk	
< 1.66%	401 (54.3)	33 (54.1)	434 (54.3)
≥ 1.66%	338 (45.7)	28 (45.9)	366 (45.8)
All	739 (100)	61 (100)	800 (100)

Kappa = 0.00; *p* = 0.98

The data show the frequencies and percentage in the parenthesis

Table 4 shows that women at high lifetime risk, 44.0% times under-estimated their risk while low or average-risk subjects, 45.0% of times over-estimated their risk in BC development. Based on actual lifetime risk, 225 (28.1%) subjects were labeled as high risk but this figure for perceived risk was 385 (48.1%) individuals. The Kappa statistics were 0.09 (*p* = 0.005) which shows a low level of agreement between perceived risk and actual risk (Kappa = 0.09, *p* = 0.005).

**Table 4:** The self-perceived lifetime risk of BC development according to corresponded estimated actual lifetime risk

Perceived lifetime risk	Actual lifetime risk		All
	< 10% risk	≥ 10% risk	
< 10%	316 (55.0)	99 (44.0)	415 (51.9)
≥ 10%	259 (45.0)	126 (56.0)	385 (48.1)
All	576 (100)	225 (100)	800 (100)

Kappa = 0.09; *p* = 0.005

The data show the frequencies and percentage in the parenthesis

Table 5 also presents that among women with low/average risk, the percentage of over-estimate of perceived risk was significantly higher among younger (*p* = 0.05), the high level of knowledge of risk factors (*p* = 0.002) and symptoms and signs (*p* = 0.004) were associated with the higher education level (*p* = 0.04). While in a high-risk group, the data did not show a clear pattern of under-estimate of perceived risk with individuals’ characteristics.

**Table 5:** The distribution of over-estimate and under-estimate with respect to individuals’ characteristics of women among low/average risk and high risk groups

Characteristics	Low/average risk (n = 739)			High risk (n = 61)		
	Accurate n (%)	Over-estimate n (%)	*p* value	Accurate n (%)	Under-estimate n (%)	*p* value
Age group (year)			0.05			0.63
35–49	241(49.9)	242(50.1)		3(33.3)	6(66.7)	
50–64	125(56.8)	95(43.2)		14(45.2)	17(54.8)	
Relatives ≥ 65	24(66.7)	12(33.3)		11(52.4)	10(47.6)	
Breast cancer at 1st degree			0.62			0.84
Relatives				16(47.1)	18(52.9)	
None	376(53.0)	334(47.0)		12(44.1)	15(55.6)	
Yes	14(48.3)	21(51.2)				
No of biopsy			0.59			0.91
None	370(53.0)	328(47.0)		20(45.5)	24(54.5)	
≥ 1	20(48.8)	21(51.2)		8(47.1)	9(52.9)	
Knowledge of risk factors			0.002			0.37
Low	221(58.3)	158(41.7)		20(50.0)	20(50.0)	
High	167(46.9)	189(53.1)		8(38.1)	13(61.9)	
Knowledge of symptoms and signs			0.004			0.17
Low	155(59.8)	104(40.2)		5(31.2)	11(68.8)	
High	230(48.8)	241(51.2)		23(51.1)	22(48.9)	
Education			0.04			0.65
Illiterate	47(69.1)	21(30.9)		6(60.0)	4(40.0)	
Elementary	78(53.1)	69(46.9)		2(28.6)	5(71.4)	
High school	182(50.3)	180(49.7)		14(45.2)	17(54.8)	
University level	83(51.2)	79(48.8)		6(46.2)	7(53.8)	
Occupation			0.12			0.75
Housewife	292(52.7)	292(47.3)		18(46.2)	21(53.8)	
Employee	93(55.4)	75(44.6)		4(57.1)	3(42.9)	
Retired	5(29.4)	12(70.6)		6(40.0)	9(60.0)	
1st menstrual age			0.09			0.47
< 12 year	48(45.3)	58(54.7)		7(53.8)	6(46.2)	
≥ 12	335(54.0)	285(46.0)		20(42.6)	27(57.4)	

The *p* values were calculated using Chi-square test

**Fig. 1:**
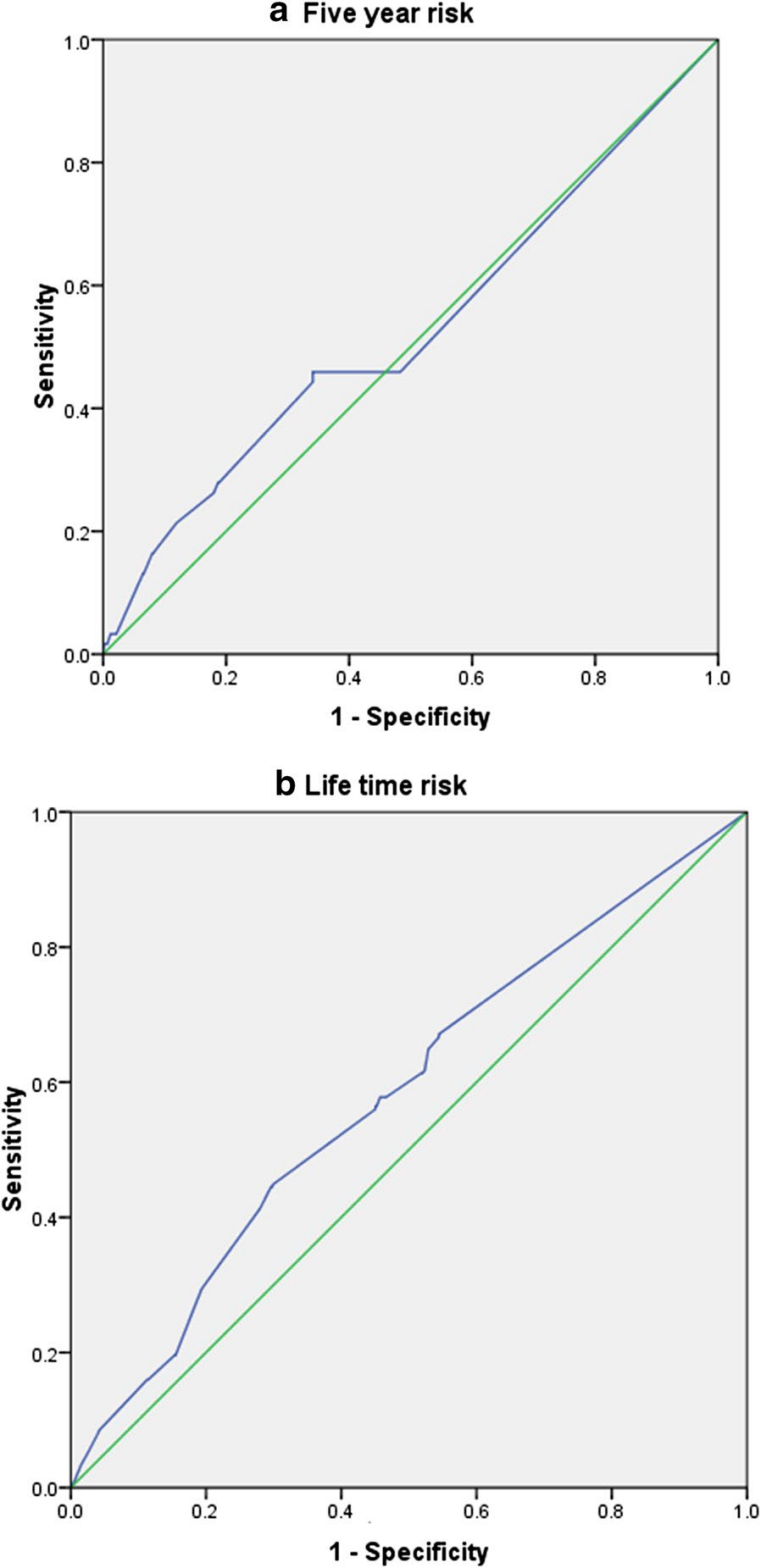
ROC curves of perceived risk by visual analog scale for prediction of actual risk (**a** for 5 year risk and **b** for lifetime risk)

Table 6 and Fig. 1 in panel (a) and (b) display the corresponded accuracy and the location of ROC curves of perceiving risk in the prediction of actual 5-year and lifetime risk. The AUCs were 0.53 (95% CI 0.44–0.61; *p* = 0.47) and 0.58 (95% CI 0.54–0.62, *p* = 0.001) for perceived risk in prediction of actual 5-year risk and lifetime risk respectively. The perceived risk has appeared with very low sensitivity and specificity (Sen = 45.9% and Sp = 54.3% for 5-year risk and Sen = 56%, Sp = 55% for lifetime risk). These findings also show the low performance for accuracy of self-perceived risk on VAS in the prediction of the actual risk. Additionally, the perceived risk in VAS yielded low performance in positive predicted value (PPV) but relatively high in negative predicted value (NPV).

**Table 6:** The performance of accuracy of self-perceived risk in BC development compared with actual risk

Perceived risk	AUC (95% CI)	*p* value	Sen (%)	Sp (%)	PPV (%)	NPV (%)
5-year risk	0.53 (0.44–0.61)	0.47	45.9	54.3	7.6	92.4
Lifetime risk	0.58 (0.54–0.62)	0.005	56.0	55.0	32.7	76.1

*AUC* area under the curve, *Sen* sensitivity, *Sp* specificity, *PPV* positive predicted value, *NPV* negative predicted value

## Discussion

The study was conducted to assess the perceived risk of breast cancer compared to the actual risk in 800 Iranian women. Finding of the study showed that the perceived risk of breast cancer in women with low and high risk of breast cancer was significantly higher than the actual risk. In other words, study women overestimated of breast cancer risk than routine. This finding indicates a significant pessimism in breast cancer perception.

The results of the study were in line with the reports of Ceber et al. [21], John and et al. [23], and Aduayi and et al. [27]. Jones and colleagues found that the estimated risk among 3000 Australian women was higher than the real risk [23]. However the results of the study were inconsistent with the findings of studies by Chung et al. [20] and Katapodi et al. [29]. A study by Chung et al. [20] found that most Korean women (51%) were optimistic about their relative risk of breast cancer and only 2.3% had pessimistic perception. In the study of Katapodi and et al. [29] an optimistic and pessimistic perception of the risk of breast cancer was 40% and 10%, respectively. Optimistic means that fewer negative events and more positive events happen to themselves than others [20]. There are probably several reasons for this inconsistency. In Chung et al. study, samples were selected from health centers. Whether, in our study samples were selected from the community and healthcare setting; it plays a role in overestimation of breast cancer risk [31]. Selection of studies’ sample from a healthcare setting such as a primary care, a hospital, or a genetic counseling clinic may be pessimism bias of breast cancer perceived risk. However, researchers also state an optimistic bias for the perceived risk had their samples from the community [31]. Health workers (physicians, midwives and nurses) that encountered with the reality of the deficiency of the pessimism opinion of the women in their daily practice can be effective to pessimism bias [31]. Future studies with comparative perceive risk of breast cancer in samples from community and health centers can be provide better information.

Also pessimism of breast cancer perceived risk can be attributed to the culture of the society [20] and women worry and anxiety [32]. Researchers report that culture is an important factor in women's perceptions of breast cancer risk [20] as well as women worry and anxiety [32]. Additionally perceive measuring tools, and women's knowledge of breast cancer risk factors [20] may also be effective. Overestimated the risk of breast cancer can be a barrier to screening for health behaviors [23, 27, 28]. The probable justification for this approach bases on theory and research, it is that if the risk, (the perceived sensitivity), is high and the condition involved (breast cancer), is considered serious, the person will do less of the recommended interventions. This action is due to high fear. Overestimate the risk of breast cancer in a person, can be lead to anxiety and it is followed by the barrier of mammography [23].

Perception of the risk of disease is the main stimulus of health behaviors to prevent, diagnose, and control cancer [30]. Aligning a person’s perceived and real risk of breast cancer leads to a more realistic understanding of risk [29]. As a result, it can be a motivator for appropriate health behaviors [29]. Women's optimism and pessimism about the risk of breast cancer make them evaluate their risks less and more and also it can prevent proper health behaviors [20].

The findings of the current study demonstrated that overestimated risk in younger and high education women was significantly higher than older women and lower levels of education. Jones et al. [33] in their study illustrated that younger age and higher education in women are two predictive factors in the overestimate risk of breast cancer. Also in another study, women between the ages of 30 and 39 years estimated the risk of breast cancer significantly higher than women older than 40 years [23]. More media focus on young women with breast cancer could be a possible reason for the increased perception of risk in this age group [23]. Another possible justification is the high incidence of breast cancer in younger women in recent years [27]. Breast cancer in younger women is associated with higher involvement and poor prognosis [34]. The incidence of breast cancer in Iranian women is 10 years lower than developed countries [35].These factors can justify the overestimate of the risk of breast cancer in younger women. In contrary to our observations, David et al. [34] and Cyrus-David et al. [36] reported women with higher education compared to lower education, have a more accurate estimate of the risk of breast cancer. The same is expected. But sometimes maybe a high level of awareness about a disease can give the opposite result.

## Limitations

This study has limitations. Our participants did not include women < 35 years old; therefore, we cannot generalize our findings to this younger. The real risk was calculated based on the Gail model that the baseline risk was adopted from US women. Perhaps their breast cancer risk is higher than Iranian women. Furthermore, the self-report risk perception scale is a subjective measure that may be potentially susceptible to be more pessimistic culturally.

## Conclusion

Women in the study overestimated their breast cancer risk higher than real risk. This finding illustrated a significant pessimism in breast cancer perception. Also overestimated risk in younger and high education women was significantly higher than older women and lower levels of education. Women at low or average risk of breast cancer need to have an accurate perception of their risk to avoid unnecessary anxiety and treatment. Therefore, increasing women’s knowledge to perception their true risk of breast cancer should be considered by health providers.

## Data Availability

To keep patients' confidentiality, the raw data would not be shared. But, it is available from the corresponding author on reasonable request, and the summary data are available in the main document.

## Abbreviations

BC: Breast cancer
VAS: Visual analog scale
HBM: Health belief model
AUC: Area under the curve
Sen: Sensitivity
Sp: Specificity
PPV: Positive predicted value
NPV: Negative predicted value
